# Soil C-N-P pools and stoichiometry as affected by intensive management of *camellia oleifera* plantations

**DOI:** 10.1371/journal.pone.0238227

**Published:** 2020-09-04

**Authors:** Liangying Liu, Ling Zhang, Jun Pan, Jiehui Niu, Xinyue Yuan, Sizhe Hu, Chunmei Liu, Nasir Shad, Jiahui Huang, Bangliang Deng, Wenping Deng, Xiaojun Liu, Wenyuan Zhang, Yuanqiu Liu

**Affiliations:** Jiangxi Provincial Key Laboratory of Silviculture, College of Forestry, Jiangxi Agricultural University, Nanchang, China; Sichuan Agricultural University, CHINA

## Abstract

Intensive management of *C*. *oleifera* has produced many pure *C*. *oleifera* plantations. The transmission of *C*. *oleifera* plantation will potentially affect soil C, N, and P pools as well as their stoichiometric characteristics both in top soil layer and vertical soil profile due to the intensive management. To understand changes in vertical pools and stoichiometric characteristics of soil C, N, and P as affected by intensive management of *C*. *oleifera* plantations, both mixed and pure *C*. *oleifera* plantations were studied. We conducted studies in five locations in Jiangxi, China with both pure and mixed *C*. *oleifera* plantations, to compare changes in vertical pools and stoichiometry of C, N, and P. Both C and N pools were significantly different between mixed and pure plantation types of *C*. *oleifera*. However, the ratio of C:N, C:P, and N:P was consistently higher in mixed plantations with C:P and N:P altered but C:N ratio did not change with soil depth. The intensive management significantly impact both C and N pools and the stoichiometry of C, N, and P. Intensive management of *C*. *oleifera* plantations decreased both C and N pools, especially at the depth of 30–50 cm soil layer. *C*. *oleifera* plantation alteration from mixed to pure should be considered in future forest management practice considering the substantial effects on soil element cycling and distribution along vertical soil profile.

## Introduction

Soil carbon (C), nitrogen (N), and phosphorus (P) are three functionally important elements associated with ecosystem nutrient cycling process and stability [1, 2]. While soil C pools account for substantial component of global C, slight changes in soil C pools may generate substantial alterations in global C distribution [2]. Nitrogen and P are two vital nutrients regulating plant growth and vegetation distribution [3]. The stoichiometric ratio of C, N, and P has been widely studied and considered as an important index reflecting ecosystem function and development [1, 2, 4]. Thereby, understanding the distribution of C, N, and P as well as their stoichiometric ratio will further our understanding of ecosystem function and stability, benefitting future management and exploration of both natural and human disturbed ecosystems [1].

Intensive management of forests or plantations have been widely implemented across the world [5]. Intensive management will generally enhance biomass accumulation [5], alterations in microbial community compositions [6, 7], increases in soil C, N, and P pools [7], and potentially alter the stoichiometric ratios. The stoichiometric ratio of C, N, and P could be influenced by vegetation, soil types, and disturbance [1, 8]. In general, the vertical distributions of C, N, and P along soil profiles experience effects of all factors mentioned above via root depth, aeration, soil moisture, fertilization or tillage [9]. Changes in vertical distribution of C, N, and P concentrations will be followed by changes in both pools and stoichiometric ratio of C, N, and P [9, 10]. Therefore, changes in vegetation types or plantation management will generally lead to altered C, N, and P pools or their stoichiometric ratio in vertical distribution along soil profile [9]. Changes in soil C, N, and P pools or their stoichiometric ratio will impact the sustainable development of natural forests or artificial plantations via changes in soil elements balance and nutrient availability or even microbial community compositions [10].

While most studies focused on forests producing timber or providing service [11], the intensive management of woody oil plant, *Camellia oleifera*, which could produce high-quality edible oil from its fruit has not been thoroughly studied [12–14]. The main distribution area of *C*. *oleifera* is red soil area in subtropical China, especially Jiangxi and Hunan province [12, 14]. Plantations of *C*. *oleifera* could be managed in hilly areas with lower nutrients that are not suitable for development of agricultural farmland [14, 15]. The development of *C*. *oleifera* plantation could thereby increase land use efficiency and provide edible oil, increasing both living standard and income of local citizens [15]. Cultivation of *C*. *oleifera* has been strongly supported and encouraged by the government in the past twenty years, increasing the distribution area of *C*. *oleifera* up to three million ha [15]. The intensive management of *C*. *oleifera*, especially fertilization, will impact soil C, N, and P balance by alterations in soil elements input [1, 3]. Compared with the natural wild plantations, intensively managed *C*. *oleifera* plantation received more N and other nutrients input and soil disturbance by tillage, which potentially impact both pools and stoichiometric ratio of soil C, N, and P along soil profile. Among all soil C, N, and P characteristics, the vertical distribution of pools and stoichiometric ratio of C, N, and P in agricultural soils contribute significantly to global element cycling and hence global change. However, no study has tried to examine changes in both pools and stoichiometric ratio of C, N, and P along vertical soil profile as affected by intensively management of *C*. *oleifera* plantations [9].

Here, we investigated both pools and stoichiometric ratio of C, N, and P along vertical soil profile as affected by intensive management of *C*. *oleifera* plantations in Jiangxi province. We aimed to answer the following questions: (1) How do pools and stoichiometry of C, N, and P change along soil profile in *C*. *oleifera* plantations? (2) How do intensive management and soil depth interact impacting pools and stoichiometric ratio of C, N, and P in *C*. *oleifera* plantation soil?

## Materials and methods

### Study sites

This study was conducted across Jiangxi province (113˚34ʹ36ʺ~118˚28ʹ58ʺE, 24˚29ʹ14ʺ~30˚04ʹ41ʺ N), where is the main distribution area of *C*. *oleifera* plantations (S1 Table). Jiangxi province lies in south part of China, characterized by subtropical climate. Annual average precipitation here is 1341–1943 mm, and annual mean temperature ranges from 16.3 to 19.5°C. Most soils are classified as red soil which are highly weathered and low in available N and P nutrients.

*Camellia oleifera* plantations haven been cultivated in Jiangxi province for decades. Both intensively managed and naturally existed *C*. *oleifera* plantations could be found from north to south in Jiangxi province. Presently, the distribution area of *C*. *oleifera* plantations is estimated to be 0.75 million ha, which represents around a quarter of the total area of *C*. *oleifera* plantations in China. Thereby, this area could be used as an ideal place studying the effects of *C*. *oleifera* plantation management on soil C, N, and P dynamics.

### Experimental design and sample collection

We investigated the distribution of both pure and mixed *C*. *oleifera* plantations across Jiangxi province from south to north (S1 Table). Within each sampled plantation, we collected soil samples by circular soil cutter from vertical soil profile of 0–10 cm, 10–20 cm, 20–30 cm, 30–50 cm, and 50–100 cm layers from November 2017 to January 2018. All plots were publicly owned and no specific permissions were required. Soil thickness was measured from soil surface after removing covered plant litter or other materials not belong to soil. We also collected soil samples by circular soil cutter with known volume from each layer to obtain soil bulk density using dry soil weight. We collected three samples as replication within each plantation type. At each studied site, two to eleven plantations were used depending on the distribution, details are shown in S1 Table. Soil sample used for C, N, and P measurements was processed by removing stones, passed through 2-mm sieve, and visible plant roots, and air-dried for use.

### Soil C, N and P measurement and pools calculation

Air-dried soil samples that had been passed through a 2 mm sieve were further ground to pass through a 0.149 mm sieve for the determination of organic C, total N, and total P [16, 17]. Soil organic C was determined by the potassium dichromate (H_2_SO_4_-K_2_Cr_2_O_7_) oxidation method [18, 19]. The H_2_SO_4_-HClO_4_ digestion method an automatic discrete chemical analyzer (Smart Chem 200, Westco, Italy) was used for analysis of N and P [20]. We calculated soil C, N and P stoichiometric ratio using dry weight basis concentrations and obtained C, N, and P pools using concentration, soil bulk density and soil layer thickness [21]. Related data was included in supporting files (S2 Table).

### Data analyses

We conducted two-way analysis of variance (ANOVA) to analyze the dependence of soil C, N, and P concentrations on *C*. *oleifera* plantation stand types and soil depth. We used the same method to analyze dependence of soil C, N, and P stoichiometric ratio as affected by *C*. *oleifera* plantation stand types and soil depth. We used post-hoc means comparisons to compare differences in C, N, and P concentrations or their stoichiometric ratios between *C*. *oleifera* plantation stand types when significant effects of plantation types were observed.

All statistical analyses were conducted by JMP 9.0 (SAS Institute, Cary, NC, USA).

## Results

### Soil C, N and P pools

Both soil C and N pools were significantly affected by *C*. *oleifera* plantation stand types, while soil P pools were not influenced (Table 1). Similarly, soil C and N pools were also impacted by soil depth, which means soil C and N pools changed along the investigated vertical soil profile (Table 1). Soil P pools did not differ along vertical soil profile either (Table 1).

**Table 1:** Soil C, N, and P pools (t ha^-1^) in *C*. *oleifera* plantation as affected by plantation types (plantations), soil depth and their interactions in two-way analysis of variance.

Factor	df	C pools			N pools			P pools		
		SS	F	P	SS	F	P	SS	F	P
Plantations	1	757	19	<0.0001	6	12	0.0006	0.00	0.01	0.9032
Soil depth	4	1684	11	<0.0001	10	5	0.0005	0.01	0.06	0.9925
Interaction	4	189	1	0.3174	0	0	0.9834	0.00	0.04	0.9968

**Fig 1:**
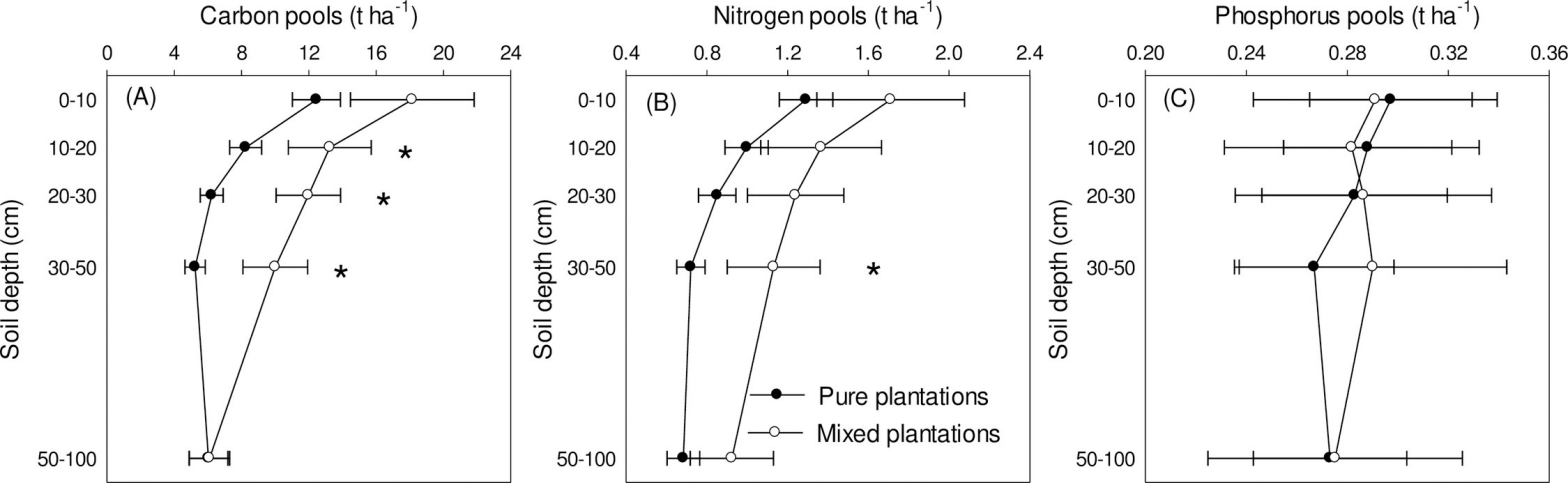
Changes in soil C, N, and P pools (t ha^-1^) as affected by *C*. *oleifera* plantation types and soil depth. Asterisks indicate significantly different between pure and mixed *C*. *oleifera* plantation types at the corresponding depth in *post-hoc* tests.

Specifically, both soil C and N pools were significantly decreased by intensively managed *C*. *oleifera* plantation, which were generally lower in pure plantation (C pools, 11.9 *vs*. 7.6 t ha^-1^ for mixed and pure plantations, respectively; N pools, 1.3 *vs*. 0.9 t ha^-1^ for mixed and pure plantations, respectively; Fig 1). Soil C pools were significantly lower at 10–20 cm, 20–30 cm, and 30–50 cm soil depth along vertical soil profile. The difference in soil N pools was observed at 30–50 cm depth between pure and mixed *C*. *oleifera* plantations (Fig 1). Soil P pools did not differ at any of the soil depth investigated (0.28 t ha^-1^ for both plantation types; Fig 1).

### Soil C, N and P stoichiometric ratio

Soil C, N and P stoichiometric ratio, including C:N, N:P, and C:P were significantly affected by plantation types (Table 2). While C:N was not differ among soil depth, both N:P and C:P were also impacted by soil depth (Table 2).

**Table 2:** The ratio of soil C, N, and P stoichiometry in *C*. *oleifera* plantation as affected by plantation types (plantations), soil depth, and their interaction in two-way analysis of variance.

Factor	df	C/N			C/P			N/P		
		SS	F	P	SS	F	P	SS	F	P
Plantations	1	288	10	0.0017	9243	24	< .0001	45	19	< .0001
Soil depth	4	104	1	0.4550	16994	111	< .0001	90	9	< .0001
Interaction	4	111	1	0.4203	1870	1	0.3104	1	0	0.9877

**Fig 2:**
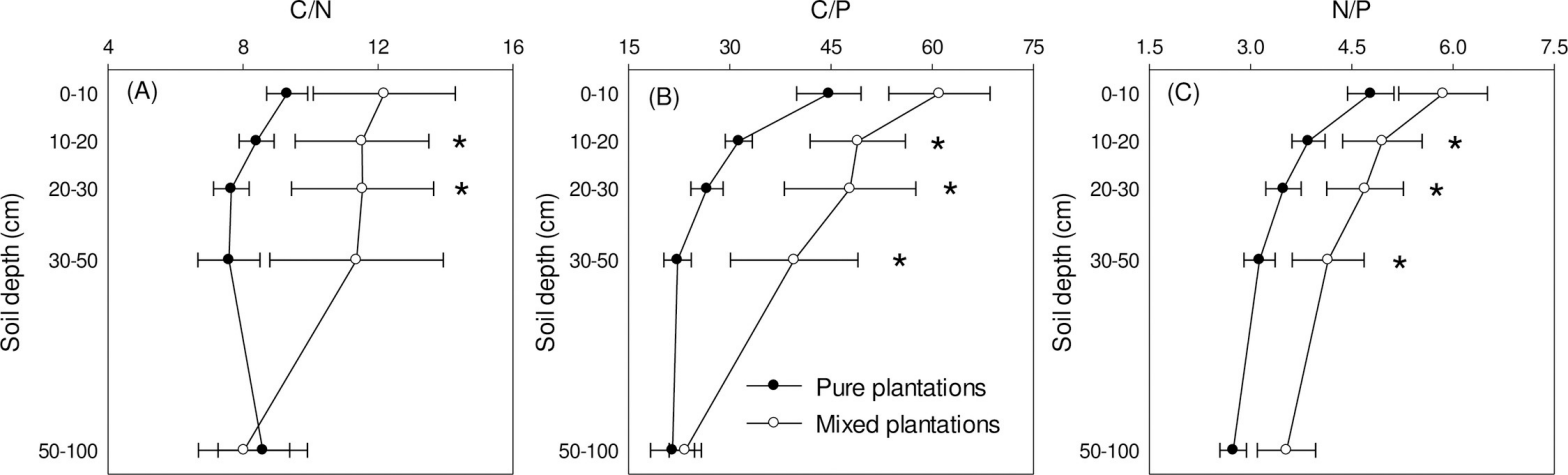
Changes in soil C, N, and P stoichiometry as affected by *C*. *oleifera* plantation types and soil depth. Asterisks indicate significantly different between pure and mixed *C*. *oleifera* plantation types at the corresponding depth in *post-hoc* tests.

Specifically, ratios of C:N, N:P, and C:P were all different at the depth of 10–20 cm and 20–30 cm, with N:P and C:P were also different at 30–50 cm depth (Fig 2). Except C:N, both N:P, and C:P showed decreasing trend along soil depth (Fig 2).

## Discussion

Carbon, N and P pools in soil as well as their stoichiometric characteristics play important role in maintenance of ecosystem function and stability [1]. Anthropogenic activities including fertilization, forest or plantation management will impose substantial effects on soil C, N, and P cycling process, balance and stoichiometry [1, 22]. Due to the great size of soil C, N, and P pools in the world, slight changes in their pools will generate significant alterations in element cycling [1]. Under the context of global change, it is crucial to understand changes in soil element cycling process as effected by intensive anthropogenic disturbance [1, 2, 23].

### Changes in soil C, N and P as affected by plantation types

The alteration in both C and N status by intensive management of *C*. *oleifera* plantation indicated potential impact on C and N content in underneath soils [22]. As an important non-timber woody oil plant, *C*. *oleifera* has been widely planted and intensively cultivated in subtropical China [12, 13]. Intensive management practice in *C*. *oleifera* plantations generally including tillage, grass control, fertilization, thinning or removing other tree or grass species [14, 15, 22], converting *C*. *oleifera* plantations from mixed plantations to pure plantations. Compared with the mixed plantations, pure *C*. *oleifera* plantations are generally sensitive to disturbance such as drought, plant disease or insect pests. Thereby, the intensively managed plantations will generally need more input of fertilization, insecticide or even irrigation. All of these practices will substantially impact materials input into *C*. *oleifera* plantation soil, which will be consequently followed by altered C, N, and P pools [14].

The changes in soil C and N pools as affected by *C*. *oleifera* plantation types might have effects on atmospheric composition [14, 24]. Due to the great size in stocking C and N, soil ecosystems are extremely important in regulating atmospheric composition, such as concentrations of carbon dioxide, nitrous oxide in the atmosphere. Both carbon dioxide and nitrous oxide are important components of greenhouse gases associated with global warming and other climate change [16, 17, 25]. The significantly decreased C and N pools in intensively managed pure *C*. *oleifera* plantation suggested more C and N loss from soil during the conversion by intensive management (Fig 1A and 1B), which might negatively impact the C and N sequestration ability of non-timber field soil and the mitigation of global climate change [26]. Thereby, mixed plantation of *C*. *oleifera* might benefit mitigation of greenhouse gas emissions more than intensively managed pure *C*. *oleifera* plantation. In future management practice of *C*. *oleifera*, this should be considered to increase soil C and N sequestration ability and mitigation of global climate change [2, 23].

Due to tight stoichiometric correlations between C, and N, the C:N ratio was significantly influenced by plantation type of *C*. *oleifera* (Table 2 and Fig 2). In general, C:N ratio was an index used to reflect microbial activities or microbial compositions [27, 28]. Higher and lower soil C:N ratios were usually associated with more fungus and bacteria, respectively [20]. Thereby, the changes in plantation composition from mixed to pure *C*. *oleifera* plantations might have altered soil microbial community compositions by increasing bacterial communities (Fig 2A), but this need to be examined in future studies.

However, soil P pools did not differ between pure and mixed *C*. *oleifera* plantations (Table 1 and Fig 1C), indicating changes in species composition and management practice of *C*. *oleifera* plantations did not influence soil P concentrations. The main distribution area of *C*. *oleifera* is subtropical area with highly weathered soil and extremely low soil nutrients, especially P [29]. The shift of *C*. *oleifera* plantation types from mixed to pure altered plant species composition substantially. However, P as one important major nutrient for plant growth, the immobilization of P in acid soil by Fe^3+^ or Al^3+^ is substantial [30, 31]. Changes in plant composition may impose slight effects on uptake of P from soil, but minimal on total P along the distribution profiles due to the easily immobilization characteristics. Interestingly, both C:P and N:P ratios were significantly different between pure and mixed *C*. *oleifera* plantations (Table 2 and Fig 2), indicating changes in soil C and N as affected by *C*. *oleifera* plantation types also exerted effects on the stoichiometric correlations between C, N, and P via ratios. Changes in N:P ratio indicating potential variations in element limitation for plant growth [4, 8, 32]. In this study, the changes in N:P ratio was not as large as that could be used as index of element limitation [8], but prolonged study will be necessary to examine the scale of alteration with time.

Thereby, shifts from pure to mixed *C*. *oleifera* plantations during intensive management have potentially alter soil C, N, and P status. Considering the vital role played by C, N, and P stoichiometry in forest ecosystems, intensive management of *C*. *oleifera* should be considered when its effects on element cycling will be evaluated.

### Changes in soil C, N and P as affected by soil depth

Nitrogen and P are the main limiting elements of plant growth in terrestrial ecosystems, their concentration and stoichiometric ratios played important role in plant growth and ecosystem function [33]. In general, soils with N:P ratio lower than fourteen are not able to provide sufficient N for plant growth, while those with N:P ratio above sixteen suggest P limitation [8]. In this study, N:P ratios in both plantation types were lower than seven point five, which indicted potential N limitation for plant growth in the investigated area [4]. Thereby, compared with the lower soil P availability in growth area of *C*. *oleifera*, intensive management might have resulted in further N limitation for plant growth.

Both C and N pools showed significant difference between pure and mixed *C*. *oleifera* plantations at the depth of 30–50 cm (Fig 1A), indicating effects on C and N occurred at this depth. While fertilization practice in the intensively managed *C*. *oleifera* plantation was usually conducted to this depth, fine root system of was mainly distributed within this layer. However, both C and N pools were decreased by intensive management of *C*. *oleifera* (Fig 1A and 1B), indicating negative effects by intensive management of *C*. *oleifera* plantations. Since soils are main sources of carbon dioxide and nitrous oxide, the decrease in soil C and N pools may generate higher C and N concentrations in atmospheric environment by increasing the concentration of carbon dioxide and nitrous oxide [12, 17, 25]. Under the context of global change, carbon dioxide and nitrous oxide increase in atmosphere will increase global temperature via global warming, resulting in series of global change events [27, 34, 35]. Thereby, the expanding intensive management of *C*. *oleifera* plantation in subtropical area might have impacted C and N exchange at the soil-atmosphere interface, which should be examined by prolonged *in situ* studies in the future and the results will benefit sustainable development of *C*. *oleifera* industries in the world.

## Conclusions

The intensive management of *C*. *oleifera* shifting mixed *C*. *oleifera* plantation to pure *C*. *oleifera* plantation significantly impact both C and N pools and the stoichiometric correlations between each pair of C, N, and P, while P pools were not influenced by *C*. *oleifera* plantation types and soil depth. Intensive management of *C*. *oleifera* plantations decreased both C and N pools at the depth of 30–50 cm soil layer, indicating potential effects on atmospheric C and N compositions. Under the context of global climate change, *in situ* study of changes in C and N efflux at the soil-atmosphere interface will be needed to understand the effects of intensive management of *C*. *oleifera* on carbon dioxide and nitrous oxide from intensively managed *C*. *oleifera* fields.

## Supporting information

S1 Table The characteristics of the sampling area used for the study. Pure plantation indicates plantations with intensive management, mixed plantation indicates plantations without intensive management.
(DOCX)

S2 Table Soil C-N-P pools and stoichiometry in both pure and mixed *Camellia oleifera* plantations. (TXT)
