# Supplementary material for: Soil C-N-P pools and stoichiometry as affected by intensive management of camellia oleifera plantations
Source: PLoS One. 2020 Sep 4;15(9):e0238227. doi: 10.1371/journal.pone.0238227 (PMC7473526; doi:10.1371/journal.pone.0238227)
Supplement: S1 Table — Pure plantation indicates plantations with intensive management, mixed plantation indicates plantations without intensive management. (DOCX) [file pone.0238227.s001.docx]

**S1 Table.** The characteristics of the sampling area used for the study. Pure plantation indicates plantations with intensive management, mixed plantation indicates plantations without intensive management.

| Location | Plantation types | Latitude | Longitude | Species composition | Canopy  density | Average Height  (m) | DBH  (diameter at breast height) | Density  (ha^-1^) |
| --- | --- | --- | --- | --- | --- | --- | --- | --- |
| Ji’an | Pure | 27º02'26" | 115º38'19" | *Camellia oleifera* | 0.6 | 1.59 | 3.65 | 2075 |
| Ji’an | Pure | 27º24'30" | 115º34'30" | *Camellia oleifera* | 0.5 | 1.49 | 2.89 | 1875 |
| Ji’an | Pure | 27º24'30" | 115º34'30" | *Camellia oleifera* | 0.6 | 1.54 | 4.15 | 2250 |
| Ji’an | Pure | 26º22'13" | 114º38'11" | *Camellia oleifera* | 0.65 | 2.13 | 8.52 | 1675 |
| Ji’an | Pure | 26º26'21" | 114º18'30" | *Camellia oleifera* | 0.55 | 2.4 | 9.24 | 1564 |
| Ji’an | Pure | 26º15'36" | 114º35'55" | *Camellia oleifera* | 0.8 | 2.10 | 7.86 | 1388 |
| Ji’an | Pure | 27º24'12" | 115º34'01" | *Camellia oleifera* | 0.8 | 2.65 | 8.86 | 1375 |
| Ji’an | Pure | 27º24'12" | 115º34'01" | *Camellia oleifera* | 0.85 | 2.41 | 8.51 | 1325 |
| Ganzhou | Pure | 25º50'39" | 114º09'30" | *Camellia oleifera* | 0.7 | 2.34 | 4.87 | 2163 |
| Ganzhou | Pure | 25º50'48" | 114º10'17" | *Camellia oleifera* | 0.9 | 2.18 | 5.1 | 2438 |
| Ji’an | Pure | 27º30'02" | 115º26'15" | *Camellia oleifera* | 0.6 | 2.61 | 4.16 | 1125 |
| Ganzhou | Mixed | 25º59'22" | 114º33'52" | *Camellia oleifera,*  *Pinus massoniana,*  *Cunninghamia lanceolata,*  *Phyllostachys edulis* | 0.8 | 8.35 | 14.96 | 1613 |
| Jiujiang | Mixed | 29º44'23" | 116º39'51" | *Camellia oleifera,*  *Liquidambar formosana, Castanopsis sclerophylla,*  *Acer truncatum Bunge* | 0.7 | 9.69 | 15.14 | 1375 |
| Jiujiang | Mixed | 29º44'13" | 116º39'33" | *Camellia oleifera, Castanopsis sclerophylla,*  *Pittosporum illicioides,*  *Lithocarpus glaber* | 0.75 | 10.27 | 16.32 | 1500 |
| Jiujiang | Mixed | 29º44'13" | 116º39'32" | *Camellia oleifera,*  *Liquidambar formosana, Castanopsis sclerophylla,*  *Lithocarpus glaber* | 0.7 | 9.68 | 15.21 | 1138 |
| Jiujiang | Mixed | 29º47'60" | 116º43'13" | *Camellia oleifera, Castanopsis sclerophylla,*  *Quercus fabri,*  *Liquidambar formosana,*  *Dalbergia hupeana* | 0.8 | 8.26 | 8.04 | 1950 |
| Jiujiang | Mixed | 29º48'02" | 116º43'13" | *Camellia oleifera, Castanopsis sclerophylla,*  *Liquidambar formosana,*  *Cunninghamia lanceolata,*  *Quercus fabri* | 0.65 | 7.87 | 7.35 | 1650 |
| Jiujiang | Mixed | 29º47'51" | 116º43'15" | *Camellia oleifera, Castanopsis sclerophylla,*  *Loropetalum chinense,*  *Dalbergia hupeana,*  *Lithocarpus glaber* | 0.7 | 9.85 | 13.88 | 2038 |
| Yichun | Mixed | 28º31'48" | 114º25'36" | *Camellia oleifera,*  *Cunninghamia lanceolata,*  *Castanopsis sclerophylla,*  *Alniphyllum fortunei* | 0.7 | 10.4 | 12.16 | 1875 |
| Yichun | Mixed | 28º29'38" | 114º21'58" | *Camellia oleifera,*  *Cunninghamia lanceolata,*  *Liquidambar formosana,*  *Photinia bodinieri* | 0.85 | 11.67 | 12.63 | 1663 |
| Yichun | Mixed | 28º32'50" | 114º20'43" | *Camellia oleifera,*  *Cunninghamia lanceolata,*  *Pinus massoniana,*  *Styrax tonkinesis* | 0.85 | 8.49 | 9.47 | 1813 |
| Yichun | Mixed | 28º32'52" | 114º20'40" | *Camellia oleifera,*  *Cunninghamia lanceolata,*  *Choerospondias axillaris ,*  *Alniphyllum fortunei,*  *Schima superba* | 0.8 | 8.94 | 9.80 | 1963 |
| Yichun | Pure | 27º43'30" | 114º11'16" | *Camellia oleifera* | 0.3 | 1.25 | 3.22 | 1025 |
| Yichun | Pure | 27º43'23" | 114º11'48" | *Camellia oleifera* | 0.3 | 1.07 | 3.36 | 1038 |
| Yichun | Pure | 27º43'25" | 114º11'42" | *Camellia oleifera* | 0.3 | 1.06 | 3.15 | 925 |
| Yichun | Pure | 27º26'34" | 114º08'08" | *Camellia oleifera* | 0.4 | 2.17 | 6.16 | 988 |
| Yichun | Pure | 27º26'39" | 114º08'04" | *Camellia oleifera* | 0.4 | 1.99 | 6.54 | 875 |
| Yichun | Pure | 27º26'40" | 114º08'06" | *Camellia oleifera* | 0.4 | 1.83 | 6.15 | 825 |
| Yichun | Pure | 27º26'28" | 114º07'56" | *Camellia oleifera* | 0.5 | 2.23 | 7.85 | 1025 |
| Yichun | Pure | 27º26'29" | 114º07'58" | *Camellia oleifera* | 0.4 | 2.12 | 7.96 | 910 |
| Yichun | Pure | 27º44'28" | 114º13'52" | *Camellia oleifera* | 0.4 | 2.12 | 6.42 | 825 |
| Yichun | Mixed | 27º48'30" | 114º09'48" | *Camellia oleifera,*  *Mallotus apelta,*  *Rhus chinensis* | 0.6 | 5.53 | 8.11 | 988 |
| Yichun | Pure | 27º48'31" | 114º09'53" | *Camellia oleifera* | 0.6 | 1.91 | 8.91 | 1025 |
| Yichun | Pure | 27º48'33" | 114º09'54" | *Camellia oleifera* | 0.65 | 2.56 | 8.80 | 1188 |
| Shangrao | Pure | 28º28'49" | 117º51'32" | *Camellia oleifera* | 0.75 | 2.48 | 6.99 | 1388 |
| Shangrao | Pure | 28º30'23" | 117º45'59" | *Camellia oleifera* | 0.7 | 1.945 | 4.10 | 1350 |
| Shangrao | Pure | 28º30'15" | 117º43'56" | *Camellia oleifera* | 0.65 | 1.85 | 4.78 | 1475 |
| Shangrao | Pure | 28º16'53" | 118º00'17" | *Camellia oleifera* | 0.65 | 1.23 | 4.44 | 2250 |
| Shangrao | Pure | 28º16'45" | 118º00'19" | *Camellia oleifera* | 0.65 | 1.19 | 4.24 | 2575 |
| Shangrao | Pure | 28º16'45" | 118º00'19" | *Camellia oleifera* | 0.65 | 1.33 | 4.49 | 2075 |
